# Physicians’ Leisure Library

**Published:** 1889-08

**Authors:** 


**Physicians' Leisure Library.** On the Treatment of the Morphia Disease. By DR. ALBRECHT ERLENMEYER. Translated by E. P. HURD, M. D. 12mo, paper, pp. 113. Twenty-five cents. Detroit, Mich.: George S. Davis. 1889.

This little book contains the gist of the chapter on Treatment in Erlenmeyer's treatise on the subject. All that is required to present his view has been given, as Dr. Hurd has chosen the practical portion of the chapter on treatment, pruning down whenever he could do so, without diminishing the value of the book.

The system of Erlenmeyer is that known as rapid withdrawal of the drug, as opposed to the gradual diminishing of the dose which sometimes extends over a period of months.

His first law is to get the patient into an institution where he can be controlled absolutely. If this is not done, he has no faith in any system of cure by any method while the patient is at liberty. Once in an institute, he begins diminishing the dose of the drug. Case IV., which he reports, may be given as a type: "Morphia habit for one and a half year's duration. Daily quantity, fifteen to twenty-two grains. Withdrawal in six days. Convalescence of twenty days."

He is a decided opponent of the system which substitutes, for the morphine, cocaine. This he denounces in the strong language which it deserves. This book is one which cannot fail to be appreciated by all who are interested in the subject; and what physician who administers morphine is not?

We can thoroughly recommend this volume as being a practical aid to the treatment of this obstinate habit. J. W. P.

